# eHealth Solutions for the Integrated Healthcare

**DOI:** 10.1155/2018/3846892

**Published:** 2018-07-10

**Authors:** Giedrius Vanagas, Rolf Engelbrecht, Robertas Damaševičius, Reima Suomi, Agusti Solanas

**Affiliations:** ^1^Lithuanian University of Health Sciences, Kaunas, Lithuania; ^2^European Federation for Medical Informatics-Health Information Management Europe ProRec, Berlin, Germany; ^3^Department of Software Engineering, Kaunas University of Technology, Kaunas, Lithuania; ^4^Department of Management and Entrepreneurship, Turku School of Economics, University of Turku, Turku, Finland; ^5^Department of Computer Engineering and Mathematics, Smart Health Research Group, Universitat Rovira i Virgili, Tarragona, Catalonia, Spain

Information and communication technologies (ICT) bring a whole new dimension into the healthcare arena by introducing electronic media that open the door to the use of new methodologies. Today, ICT support electronic gathering, storage, processing, and exchange of information to treat disease, prevent illness, promote healthy lifestyle, manage patients with chronic illness, and many other applications [1, 2]. Electronic health (eHealth) has the ability to bridge gaps between patients and doctors, patients and relatives, doctors and administrative staff, and so on. Also, eHealth helps to overcome barriers by means that are significantly different from traditional healthcare solutions [3]. It has paved the way for the adoption of sophisticated forms of healthcare provision based on mobile devices and context-awareness, such as mobile health (mHealth) [4] and smart health (sHealth) [5], which require unprecedentedly complex integration strategies.

The advances in healthcare and ICT serve the society as a whole (e.g., patients, healthcare professionals, relatives, and governments) and support the sustainability of healthcare systems. New health ICT systems offer opportunities to improve the effectiveness and efficiency of healthcare services through innovative approaches in clinical service delivery, personalized health, and public health, with a wide impact on the well-being of individuals. Clearly, the study of the integration of healthcare solutions based on ICT is a hot topic that deserves attention.

Despite the well-known potential benefits of adopting eHealth solutions, approaches to ICT implementation used in other industries have had limited success in the healthcare sector. This is not surprising, especially if we take into account the complexity and nature of the healthcare ecosystem. Hence, the implementation of ICT in practice remains difficult and implies many challenges at different levels, involving patients, healthcare providers, and healthcare organizations [6]. Some of the most significant challenges for the adoption of eHealth (and its mHealth and sHealth derivations) are inherent to the complexity of the healthcare system. Take, for example, the *interoperability* problem, which is an old problem inherited from previous nonelectronic healthcare infrastructures that is also common in a variety of other domains [7]. Also, ethical issues such as *privacy protection* are a common problem that affects healthcare in general, but it is magnified by the use of ICT [8, 9]. Last but not least, the *integration of new technologies* based on artificial intelligence, smartphones, neural networks, and big data analysis (to name a few) into the healthcare sector poses magnificent challenges for the current healthcare system's limitations, which the scientific community is struggling to overcome. In this sense, the feasibility of many applications, policies, and data concerning the costs, effects, and effectiveness of eHealth and telemedicine are at stake. There is a lack of research-based, empirically-tested models to progress towards large-scale use of ICT in the health sector.

In this special issue, we have undertaken the task of collecting a set of articles that address some of the aforementioned challenges faced by current eHealth infrastructure and applications.

Monitoring long-distance fast walking in daily activities is challenging due to the lack of specific scientific equipment. In the literature, most gait experiments are performed by walking on treadmills. Unfortunately, gait data acquired on treadmills are quite different from the real ones gotten on the ground. The article by W.-F. Wang et al. “*Study on Tripping Risks in Fast Walking through Cadence-Controlled Gait Analysis*” approaches this problem by performing all walking tests intended to reveal important clues for tripping risks in fast walking in a flat and straight pathway. The results show that fast walking with bigger strides and lower cadence is the best way to keep safety on ordinary ground.

Similarly, the monitoring of vital signs is a very active research area. In the article “*Analysis of a Pulse Rate Variability Measurement Using a Smartphone Camera*,” A. Bánhalmi et al. approach the problem of using off-the-shelf technology (e.g., smartphones) to gather biometric data efficiently and reliably. More specifically, the authors analyse the possibility of using a smartphone camera to measure the pulse rate variability of patients. Their experiments show that photoplethysmography has high accuracy and does not differ more from ECG than ECG channels themselves. This study opens the door to simplified remote monitoring of the heart function.

In a related topic, haemovigilance is attracting attention. It is the set of surveillance procedures covering the entire blood transfusion chain, from the donation and processing of blood and its components, through to their provision and transfusion to patients, and including their follow-up [10]. In the article “*The Evolving Role of Information Technology in Haemovigilance Systems*,” A. Ramoa et al. study 23 haemovigilance organizations in their use of information systems. They find an increasing number of these organizations choosing web-based solutions to take care of haemovigilance. There are still some nonelectronic notification systems, but they lack in data completeness and consistency. The authors support the development of electronic haemovigilance systems and conclude that national haemovigilance systems could benefit from international guidelines for their implementation and maintenance.

There are a number of approaches to develop functionalities for medical decision support systems, which involve some extra efforts from users, thus limiting the spread of such systems in practice. V. L. Malykh and S. V. Rudetskiy provide a review of general approaches to decision support systems development based on nonreduced big clinical data in their article “*Approaches to Medical Decision-Making Based on Big Clinical Data*.” The article discusses different approaches to building a medical decision support system based on big data. The authors sought to abstain from any data reduction and apply universal teaching and big data processing methods independent of disease classification standards. The paper assesses and compares the accuracy of recommendations among three options: case-based reasoning, simple single-layer neural network, and probabilistic neural network. Further, the paper substantiates the assumption regarding the most efficient approach to solving the specified problem.

In a similar research line, knowledge-based systems can notably improve the management of distributed eHealth systems, where communication and understanding between medical professionals and different tasks become essential. Usually, medical texts are full of references to medical entities, which could be utilized by knowledge-based medical decision support systems. However, the diverse and ambiguous nature of linguistic ME forms are challenging and hinder automatic medical entities recognition and linking, hence requiring tedious work to annotate data and define features. Xu et al. propose an unsupervised framework for recognizing and linking medical entities mentioned in Chinese online medical texts. Their solution is the first complete unsupervised solution for Chinese medical text with both medical entity recognition and linking, which has considerable value in many applications such as medical knowledge-based construction and expansion, semantic comprehension of medical text, and medical Q&A systems.

Interacting with healthcare systems and applications is an emerging topic that deserves special attention when dealing with disabled people. The article “*Projection mapping user interface for disabled people*” by J. Gelšvartas et al. seeks to help improve user interfaces for people with motoric or speech disabilities through projection mapping. This technique makes possible to create a natural augmented reality information presentation environment. The authors provide a detailed description of a camera-projector system calibration procedure. The described system performs tabletop object detection and automatic projection mapping. The proposed system was tested with real users and, overall, the interface was evaluated positively by the system users, which in most cases were able to learn how to use the system very quickly. The article clearly opens ways for new ideas to produce support systems for motoric or speech disabled people.

Last but not least, the constant aging of the population raises a new set of problems for eHealth systems. In coming years, age-related and degenerative diseases will become the main burden for public health systems. Lauraitis et al. address the problem of identifying Huntington's disease (HD) at its early stage, so that elder patients could benefit from future medical interventions that may help delaying the progress of the disease. They created a computerized behavioural model, which allows predicting an impaired reaction condition for HD patients. The model is embodied in a mobile application available on smartphones and tablet PCs, which allows predicting the functional capacity level of subjects by performing an on-screen touch-based test, thus providing a low-cost alternative to the currently used HD symptom assessment procedures.

The integration of eHealth solutions is far from simple, and it must capture the attention of the healthcare research community for the next years. The adoption of new mobile and context-aware technologies will multiply the benefits for patients and doctors but, at the same time, it will increase the complexity of the eHealth systems and it will be associated to major challenges. In this special issue, we have gathered some relevant examples of research studies that have put their efforts towards this direction. Notwithstanding, there is a lot of unused potential in information systems to support eHealth and its mHealth and sHealth derivations.

It has become clear that technology is not the limiting factor for future progress in healthcare. On the contrary, the main limitations come from the lack of innovativeness of ICT use and the lack of incentives from users and communities to adopt new technology-based solutions for the integrated healthcare. We hope that these limitations will be solved in the next years and that the articles collected in this special issue will contribute with their grain of sand to the overall improvement of healthcare systems worldwide.

*Giedrius Vanagas* *Rolf Engelbrecht* *Robertas Damaševičius* *Reima Suomi* *Agusti Solanas*
